# Stability Characterization of a Vaccine Antigen Based on the Respiratory Syncytial Virus Fusion Glycoprotein

**DOI:** 10.1371/journal.pone.0164789

**Published:** 2016-10-20

**Authors:** Jessica A. Flynn, Eberhard Durr, Ryan Swoyer, Pedro J. Cejas, Melanie S. Horton, Jennifer D. Galli, Scott A. Cosmi, Amy S. Espeseth, Andrew J. Bett, Lan Zhang

**Affiliations:** 1 Infectious Diseases and Vaccines Discovery, MRL, Merck & Co., Inc., Kenilworth, New Jersey, United States of America; 2 Eurofins Lancaster Laboratories Professional Scientific Services, Lancaster, Pennsylvania, United States of America; New York State Department of Health, UNITED STATES

## Abstract

Infection with Respiratory Syncytial Virus (RSV) causes both upper and lower respiratory tract disease in humans, leading to significant morbidity and mortality in both young children and older adults. Currently, there is no licensed vaccine available, and therapeutic options are limited. During the infection process, the type I viral fusion (F) glycoprotein on the surface of the RSV particle rearranges from a metastable prefusion conformation to a highly stable postfusion form. In people naturally infected with RSV, most potent neutralizing antibodies are directed to the prefusion form of the F protein. Therefore, an engineered RSV F protein stabilized in the prefusion conformation (DS-Cav1) is an attractive vaccine candidate. Long-term stability at 4°C or higher is a desirable attribute for a commercial subunit vaccine antigen. To assess the stability of DS-Cav1, we developed assays using D25, an antibody which recognizes the prefusion F-specific antigenic site Ø, and a novel antibody 4D7, which was found to bind antigenic site I on the postfusion form of RSV F. Biophysical analysis indicated that, upon long-term storage at 4°C, DS-Cav1 undergoes a conformational change, adopting alternate structures that concomitantly lose the site Ø epitope and gain the ability to bind 4D7.

## Introduction

Respiratory Syncytial Virus (RSV) infections are common and generally cause mild, cold-like symptoms in healthy adults and older children. However, in premature babies, infants, older adults and immunocompromised individuals, RSV can lead to more severe lower respiratory tract disease, causing pneumonia or bronchiolitis, and may be life-threatening [1–4]. Despite extensive research effort, there is no vaccine available to prevent RSV infection or disease.

Passive prophylaxis with palivizumab (Synagis®), however, is approved for use in a subset of preterm infants that are at greatest risk for developing severe RSV-induced lung disease. Palivizumab is a humanized monoclonal antibody that binds one of the RSV surface-exposed envelope glycoproteins, the fusion protein F [5, 6]. The clinical efficacy of palivizumab, a reduction in RSV-related hospitalization [7, 8], provides proof of concept that a vaccine that can elicit an anti-F neutralizing antibody response would prove effective against RSV-induced disease.

**Fig 1:**
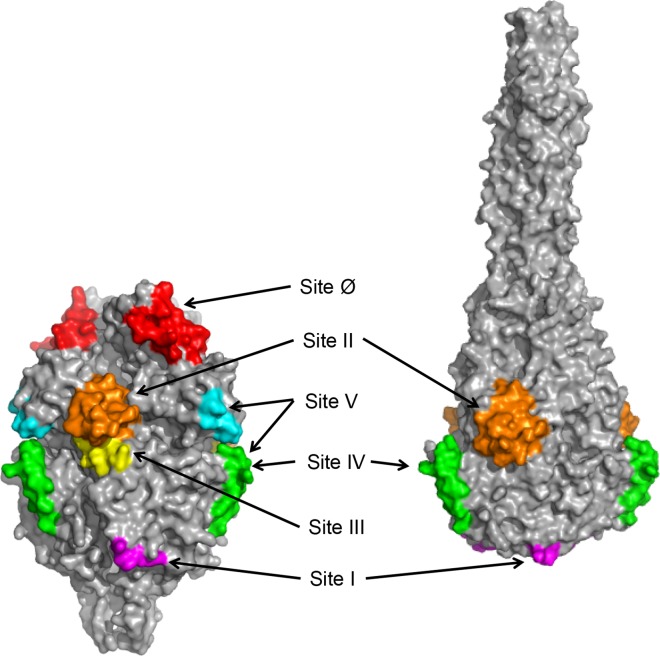
RSV F prefusion and postfusion structures and antigenic sites. Surface representation of prefusion (left panel) and postfusion RSV F (right panel) trimers are shown in gray [13, 20]. Antigenic sites are highlighted in different colors: site Ø, red; site I, magenta; site II, orange; site III, yellow. Sequences contained in the overlapping antigenic sites IV and V are green, and additional residues that contribute to site V antibody AM14 binding are shown in cyan.

Targeting RSV F as a vaccine antigen is complicated by the fact that the protein can adopt multiple conformations. On the virus surface, RSV F can exist in a metastable prefusion conformation that, during the infection process, rearranges to a more stable postfusion form (Fig 1), to enable virus entry into the host cell. At least two antigenic sites exposed on both the prefusion and postfusion forms of F (sites II and IV) are recognized by antibodies with neutralizing activity (Fig 1) [9–13]. However, depleting postfusion F-binding antibodies from convalescent human serum only modestly reduces the ability of the sample to neutralize RSV [14–16]. Adsorption of antibodies that bind the prefusion conformation of F, in contrast, removes almost all of the serum neutralizing activity [16]. Taken together, these data indicate that the majority of the neutralizing antibody response induced by natural RSV infection is directed toward epitopes specific for prefusion F. Several potent prefusion F-specific neutralizing antibodies, recognizing multiple antigenic sites, have been described previously. These include MPE8, which binds site III [17], AM14, which recognizes site V [18], and D25, which binds site Ø [19] (Fig 1).

The structure of prefusion RSV F in complex with D25 was solved by McLellan et al. [21], enabling the design and characterization of DS-Cav1, a soluble RSV F protein stabilized in the trimeric prefusion conformation by a heterologous trimerization motif (foldon), cavity filling mutations and a non-native disulfide bond [20]. DS-Cav1 binds to a panel of prefusion-specific, site Ø-directed, monoclonal antibodies, and the integrity of antigenic site Ø on DS-Cav1 is largely maintained following exposure to increasing temperature, as well as pH and osmolality extremes [20]. Immunization of preclinical animal species with DS-Cav1 elicits a robust serum neutralizing response, highlighting the potential of DS-Cav1 as a vaccine candidate [20].

Understanding the long-term conformational stability of a vaccine antigen is a requirement during vaccine development. To assess the stability of DS-Cav1 stored at 4°C, we have developed assays using antibodies that can discriminate between the prefusion and postfusion forms of RSV F. To that end, we characterized a newly identified RSV F-binding mouse monoclonal antibody, 4D7. Surface plasmon resonance (SPR) was used to demonstrate that 4D7 does not bind to site Ø-containing DS-Cav1 protein, and a shotgun mutagenesis approach was employed to map the 4D7 epitope to antigenic site I. Importantly, the SPR assay revealed that DS-Cav1 preparations contain a subset of protein that does not bind to site Ø-specific antibodies like D25, but is recognized by 4D7. DS-Cav1 preparations stored at 4°C gained 4D7 reactivity over time, and the increase in 4D7 binding was paralleled by a decrease in D25 binding. These data, together with protein visualization by electron microscopy and analysis by differential scanning fluorimetry suggest that, during prolonged storage at 4°C, DS-Cav1 shifts away from a site Ø-containing prefusion conformation to one that is distinct from both the prefusion and postfusion F forms.

## Materials and Methods

### Monoclonal antibody production and sequencing

To produce human antibodies D25, MPE8 and AM14, the heavy and light chain variable region sequences were subcloned into a pTT5 vector [17–19]. Plasmids encoding both the heavy and light chain were transiently co-transfected into suspension CHO-3E7 cells grown in serum-free FreeStyle CHO Expression Medium (Life Technologies). The supernatants collected after 6 days were applied to a Protein A CIP column (Genscript) for purification. The purified antibodies were buffer-exchanged to phosphate-buffered saline (PBS).

Mouse hybridoma cultures were generated by standard methods (Kohler, 1975). All animal studies were approved by the Merck Institutional Animal Care and Use committee, and mice were maintained in accordance with the *Guide for the Care and Use of Laboratory Animals* by the National Research Council. Briefly, BALB/c mice were infected intranasally with RSV strain A2 twice before they were boosted with 20 μg purified A2 virus (Advanced Biotechnologies, Inc.) in 100 μL PBS by intravenous tail vein injection. Splenic lymphocytes were fused with a mouse myeloma partner, cultures were screened for virus binding and the monoclonal mouse IgG2a kappa antibody 4D7 was identified. 4D7 was purified from supernatant harvested from 4D7-secreting mouse hybridoma cells by SDIX (Newark, DE) on a Protein A Sepharose Fast Flow column. Purified antibody was stored in 20 mM sodium phosphate, 150 mM NaCl at pH 7.2.

The 4D7 antibody sequence was determined at GenScript (Piscataway, NJ). To sequence 4D7, total RNA was extracted from hybridoma cells using TRIzol® reagent (Life Technologies). Extracted RNA was reverse transcribed into cDNA with isotype-specific antisense or universal primers using a PrimeScript^TM^ 1st Strand cDNA Synthesis Kit (Takara). Amplified antibody fragments were separately cloned into a standard cloning vector and sequenced.

### Production of DS-Cav1 and postfusion F proteins

As previously described, plasmids encoding mammalian codon-optimized RSV F prefusion (DS-Cav1) and postfusion (FΔFP) proteins were used to transfect Expi 293F cells (Life Technologies), and proteins were purified from culture supernatants [13, 21]. Briefly, cell culture supernatants were harvested day 3 (FΔFP) or 7 (DS-Cav1) post-plasmid transfection, and RSV F proteins were purified using Ni-Sepharose chromatography (GE Healthcare). FΔFP was further purified by Strep-Tactin chromatography (Strep-Tactin Superflow Plus, Qiagen). Tags were removed from DS-Cav1 and FΔFP by overnight digestion with thrombin. To remove IMAC contaminants and uncleaved F protein, DS-Cav1 was subjected to a second Ni-Sepharose chromatography step. Both DS-Cav1 and FΔFP were polished by gel filtration chromatography (Superdex 200, GE Healthcare) and were stored in a buffer of 50 mM HEPES pH 7.5, 300 mM NaCl.

### Epitope mapping

Shotgun mutagenesis epitope mapping for 4D7 was performed by Integral Molecular, Inc. (Philadelphia, USA). Alanine scanning mutagenesis of an expression construct for RSV F (from RSV-A2; NCBI ref # FJ614814) targeted 368 surface-exposed residues identified from the crystal structures of both prefusion and postfusion conformations of RSV F [13, 21]. Each residue of interest was individually mutated to an alanine (or alanine residues to serine).

Library screening was performed essentially as described previously [22]. Briefly, the RSV F library clones were transfected individually into human HEK-293T cells and allowed to express for 16 hrs before cells were fixed in 4% (v/v) paraformaldehyde (Electron Microscopy Sciences) in PBS containing calcium and magnesium. Cells were incubated with monoclonal antibodies diluted in 10% (v/v) normal goat serum (NGS) for 1 hour at room temperature, followed by a 30 minute incubation with 3.75 μg/mL Alexa Fluor 488-conjugated secondary antibody (Jackson ImmunoResearch Laboratories) in 10% (v/v) NGS. Primary monoclonal antibody concentrations were determined using an independent immunofluorescence titration curve against wildtype RSV F to ensure that the signals were within the linear range of detection. Cells were washed twice with PBS without calcium or magnesium and resuspended in Cellstripper (Cellgro) plus 0.1% (v/v) BSA (Sigma-Aldrich). Cellular fluorescence was detected using the Intellicyt high throughput flow cytometer (Intellicyt).

Antibody reactivity against each mutant clone was calculated relative to wildtype protein reactivity by subtracting the signal from mock-transfected controls and normalizing to the signal from wildtype protein-transfected controls. Mutations within clones were identified as critical to the monoclonal antibody epitope if they did not support reactivity of the test antibody, but supported reactivity of other antibodies, such as palivizumab. This counter-screen strategy facilitated the exclusion of RSV F protein mutants that were misfolded or had an expression defect. The detailed algorithms used to interpret shotgun mutagenesis data are described elsewhere [23].

### Surface Plasmon Resonance

The surface plasmon resonance (SPR) experiments were performed on a Biacore 2000. All experiments used HBS-EP buffer (0.01 M HEPES pH 7.4, 0.15 M NaCl, 3 mM EDTA, 0.005% v/v Surfactant P20, GE Healthcare Life Sciences) and were carried out at room temperature. Anti-mouse IgG (GE Healthcare Life Sciences) or D25 (2000 RUs of each) were amine coupled to separate channels of a CM5 sensor chip (GE Healthcare Life Sciences). Prior to flowing the analyte, 500 RUs of 4D7 antibody were captured on the anti-mouse IgG channel. The first channel was left blank for reference subtraction. Unless otherwise noted, postfusion F or DS-Cav1 was flowed over all surfaces at a concentration of 40 μg/mL for 150 s at a flow rate of 10 μL/min. For certain experiments, 3 μg DS-Cav1 was preincubated with either D25 or 4D7 (100 μg/mL) in a volume of 20 μL, and the antibody:protein mix was flowed over the D25-coupled channel after diluting with HBS-EP buffer to 20 μg DS-Cav1/mL. For sandwich SPR experiments, DS-Cav1 was flowed over the 4D7-coupled sensor chip as described above before palivizumab or D25 (40 μg/mL) was flowed over the 4D7-captured protein for 120 s. For all SPR experiments, the sensor chip surface was regenerated using two 40 second injections of 75 mM phosphoric acid at a flow rate of 30 μL/min. Response units were plotted against time, in seconds.

### Sandwich ELISA

96-well plates were coated with 0.1 μg per well of capture antibody 4D7 in PBS overnight at 4°C. Unbound sites were blocked by addition of 2% (v/v) bovine serum albumin (BSA) in PBS and incubation for 1 hour at room temperature. Following a wash step with PBS containing 0.05% (v/v) Tween 20 (PBS-T), DS-Cav1 protein stored at 4°C for approximately 5 months was 4-fold serially diluted, added to the plates at concentrations ranging from 3 ng/mL to 50 μg/mL and incubated at room temperature for 1 hour. Plates were washed with PBS-T and incubated with 1 μg/mL detection antibody (D25, palivizumab, MPE8 or AM14) at room temperature for 1 hour. Plates were washed again with PBS-T and incubated for 1 hour at room temperature with goat anti-human IgG HRP-conjugated secondary antibody (Thermo Fisher) diluted 1:2,000. Following an additional wash with PBS-T and brief rinse with ddH_2_O, Super AquaBlue ELISA substrate (eBiosience) was added, and the plate was immediately read at 405 nm for 5 min. mOD/min was calculated for each well.

### Transmission electron microscopy and image analysis

Electron microscopy and 2D class averaging were performed by NanoImaging Services, Inc. (San Diego, CA). Samples were prepared on continuous carbon films supported on nitrocellulose-coated 400 mesh copper grids (Ted Pella). A 3 μL drop of purified RSV F protein at a concentration of 2–8 μg/mL was applied to a freshly plasma-cleaned grid for 1 min and blotted to a thin film using filter paper. The sample was washed four times by floating the grid on a droplet of H_2_O for 1 min followed by staining on a droplet of 3% (w/v) uranyl formate for 1 min. The grid was blotted after each incubation and air-dried. Transmission electron microscopy was performed using an FEI Tecnai T12 electron microscope operating at 120 kV equipped with an FEI Eagle 4k x 4k CCD camera. Images were collected at nominal magnifications of 110,000x (0.10 nm/pixel), 67,000x (0.16 nm/pixel), 52,000x (0.21 nm/pixel) and 21,000x (0.5 nm/pixel) using the automated image acquisition software package Leginon [24]. Images were acquired at a nominal underfocus of -2 μm to -1 μm (110,000x), -3 μm to -1 μm (67,000x), -4 μm to -2 μm (52,000) and -4 μm (21,000x) and electron doses of approximately 9-39e/Å2.

Image processing was performed using the Appion software package [25]. Contrast transfer functions of the images were corrected using Ace2 [26]. Individual particles in the 67,000x and 110,000x images were selected using automated picking protocols, followed by several rounds of reference-free alignment and classification based on the XMIPP processing package to sort them into self-similar groups [27].

### Differential scanning fluorimetry

Analyses were performed with DS-Cav1 and postfusion F protein stored at -70°C, as well as DS-Cav1 stored for 90 days at 4°C. Solutions of F protein (0.27–35 μM) in 50 mM HEPES, 300 mM NaCl at pH 7.5 were prepared by serial dilution. The fluorescence signal of each 85 μL protein sample in a micro quartz cuvette with an optical path length of 3 mm x 3 mm (Thermo Fisher) was detected using a Cary Eclipse fluorimeter equipped with a Cary temperature controller (Agilent Technologies, CA). The intrinsic protein fluorescence was recorded at 330 nm and 350 nm. The excitation wavelength was set to 280 nm with a slit width of 10 nm. The emission slit width was set to 2.5 nm. The photo multiplier voltage was adjusted before each measurement to values between 500V and 800V to maximize the fluorescence signal. Thermal unfolding experiments were performed using a temperature ramp of 1°C/min from 20°C to 95°C in 0.5°C increments. The sample was equilibrated at the starting temperature for 1 min and fluorescence signals were averaged for each data point for 1.5 s. A multicell holder allowed analysis of up to 4 samples simultaneously. Raw data was exported for further processing with Origin Pro^®^7.5 SR7 to obtain melting curves of fluorescence intensity as a function of temperature. The melting curves were smoothed (polynomial order = 1, number of points = 12), and peak centers of the first derivative of the ratio between 350 nm and 330 nm were used as melting temperatures (Tm). The Tm presented is the mean obtained from all protein concentrations analyzed for the same sample type. Data was normalized by the highest signal intensity in order to aid the comparison of different protein samples or protein concentrations. The concentration-dependent intensity change of the low temperature transition at 60.85°C of DS-Cav1 stored at -70°C was quantified by integrating the signal of the 350 nm/330 nm melt curve between 50°C and 75°C.

## Results

### The monoclonal antibody 4D7 binds to antigenic site I on RSV F protein

The monoclonal antibody 4D7 was isolated from mice infected intranasally with RSV A2, and immunoprecipitation experiments revealed the target of 4D7 to be the RSV F protein (data not shown). The sequences of 4D7 heavy and light chain variable regions are depicted in S1 Fig. To map the epitope recognized by 4D7 on the RSV F protein, we used a shotgun mutagenesis methodology, a high-throughput cellular expression technology that enables the expression and analysis of large libraries of mutated target proteins within eukaryotic cells or on the cell surface [23]. Based on the crystal structures of the prefusion and postfusion RSV F proteins [13, 21], we identified 368 surface-exposed residues, and each was individually mutated to an alanine (or alanine to serine) to generate a comprehensive mutant RSV F expression library. For this technique, critical residues, defined as those amino acids whose side chains make the highest energetic contributions to the antibody-epitope interaction, are identified by the loss of antibody binding when these residues are mutated [28, 29]. To validate the RSV F mutant library, we first mapped the epitope of palivizumab, a well-characterized antibody which binds to both prefusion and postfusion F isoforms. In agreement with monoclonal antibody-resistant mutant and co-crystal structural studies [30, 31], we identified antigenic site II residues D269 and K272 as two critical residues for palivizumab binding (data not shown), confirming the integrity of our mutant library.

**Fig 2:**
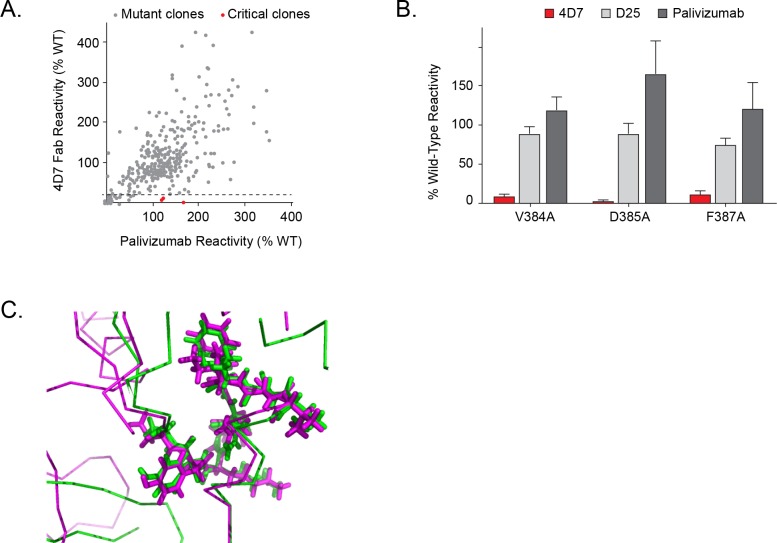
Identification of critical residues for monoclonal antibody 4D7 binding using shotgun mutagenesis epitope mapping. A shotgun mutagenesis alanine scanning library was constructed for the RSV F protein. The library contains 368 individual mutations at residues identified as surface exposed on the prefusion and postfusion forms of RSV F proteins. Each well of the mutation array plate contained one mutant with a defined substitution. (A) Human HEK293T cells expressing the RSV F mutation library were tested for immunoreactivity with 4D7, measured on an Intellicyt high-throughput flow cytometer. Clones with reactivity of <15% relative to that of wildtype RSV F (horizontal line) yet >70% reactivity for a control monoclonal antibody were initially identified to be critical for 4D7 binding (red dots), and were verified using algorithms described elsewhere [23] (U.S. patent application 61/938,894). (B) Mutation of three individual residues reduced 4D7 binding (red bars) but not the binding of D25 and palivizumab (gray bars). Error bars represent range (half of the maximum minus minimum values) of at least two replicate data points. (C) Comparison of 4D7 binding epitope on prefusion and postfusion RSV F structures. Prefusion RSV F structure is shown in magenta and postfusion F structure shown in green. Residues 384 to 392 are shown in stick representation highlighting both main chain and side chain atoms, and the rest of the structure is shown in line representation with only main chain bonds depicted.

We next compared 4D7 and palivizumab binding to each mutant clone, and three residues on the F protein (V384, D385 and F387) were identified as critical for binding to 4D7 (Fig 2A). Alanine mutations at these positions significantly reduced 4D7 reactivity but did not affect the binding of palivizumab or other control antibodies such as D25 (Fig 2B). These data indicate that the residues identified are directly involved in 4D7 binding, and the mutations introduced do not reduce RSV F surface expression levels or cause protein misfolding. The lower reactivity of D385A with 4D7 suggests that D385 is the major energetic contributor to binding, with lesser contributions made by V384 and F387.

The identified epitope is located in a short loop that contains the previously characterized antigenic site I of RSV F [10, 13]. Comparison of prefusion and postfusion RSV F structures revealed that site I adopts similar conformations in both structures (Fig 2C); however, it appears that accessibility of the site might be different. While the epitope is exposed on the top of the head domain in the postfusion F structure, it sits at the base of the head domain in the prefusion structure (Fig 1).

### The monoclonal antibody 4D7 recognizes an epitope accessible on the postfusion, but not prefusion, form of RSV F protein

**Fig 3:**
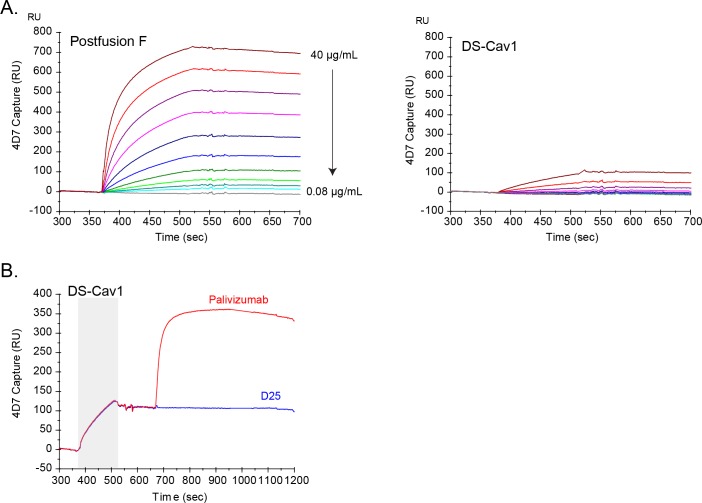
Characterization of monoclonal antibody 4D7 binding to RSV F. Surface plasmon resonance was used to assess the ability of 4D7 to bind DS-Cav1 and postfusion forms of RSV F. (A) Various concentrations of DS-Cav1 or postfusion F (two-fold serial dilutions from 40–0.08 μg/mL) were flowed over the surface of a 4D7-coated sensor chip, and response units were plotted over time, in seconds. Each line represents the results from a single concentration of postfusion F (left panel) or DS-Cav1 (right panel). (B) DS-Cav1 was flowed over the surface of a 4D7-coated sensor chip (grey shaded area), followed by palivizumab (red line) or D25 (blue line). Response units were plotted over time, in seconds.

Previous studies have suggested that antibodies directed against antigenic site I preferentially bind to the postfusion F protein [18, 32, 33]. To characterize the specific binding properties of 4D7, we used SPR to evaluate binding to both prefusion and postfusion forms of RSV F. Given the amount of ligand captured on the sensor chip surface and the relatively high concentrations of analytes used, all SPR experiments were designed to qualitatively, but not quantitatively, assess antibody binding to RSV F protein. As postfusion F protein was flowed over the surface of a 4D7-coated sensor chip, a concentration-dependent increase in SPR response units over time was observed (Fig 3A). In contrast, 4D7 captured only a small amount of the prefusion-stabilized DS-Cav1 protein, even at the highest protein concentration tested (Fig 3A). Similar results were obtained when either DS-Cav1 or the postfusion RSV F protein was coated directly on an ELISA plate and binding of 4D7 was evaluated (data not shown).

These data suggested that 4D7 at least preferentially bound the postfusion form of RSV F; however, it was unclear whether 4D7 weakly recognized an epitope present on the prefusion form of the protein or whether the DS-Cav1 protein preparation contained a mixed population of protein conformations, some of which presented a 4D7-accessible epitope. To address this question, two additional SPR experiments were done. In a sandwich SPR assay, DS-Cav1 was flowed over a 4D7-coated sensor chip, and as observed in Fig 3A, a small amount of protein was captured (Fig 3B). Subsequently, palivizumab or D25 was flowed over the 4D7:protein complex. While palivizumab was able to bind 4D7-captured protein, as indicated by an increase in SPR response units, D25, which recognizes prefusion F-specific site Ø, was unable to bind (Fig 3B). In the reverse experiment, binding of 4D7 to D25-captured DS-Cav1 was not detected (data not shown). While these data suggest that 4D7 and D25 bind independent protein species present in the DS-Cav1 preparation, it was possible that both epitopes are present on a single molecule but binding of one antibody to DS-Cav1 abrogated the ability of the second antibody to bind the same molecule. To address this, DS-Cav1 was pre-incubated with 4D7, and the mix was flowed over immobilized D25. The resulting SPR response curve overlapped that of DS-Cav1 alone, indicating that 4D7 was unable to block the binding of site Ø-containing prefusion F protein to D25 either through steric hindrance or by allosterically altering the D25 epitope (S2 Fig). Taken together, these data indicate that 4D7 binds an epitope present on postfusion F that is not present or not accessible on the site Ø-containing prefusion form of the RSV F protein. Furthermore, 4D7 is able to recognize an epitope exposed on a fraction of the DS-Cav1 antigen preparation that does not bind to D25.

### 4D7 antibody-based assay reveals DS-Cav1 conformational shift during long-term storage

**Fig 4:**
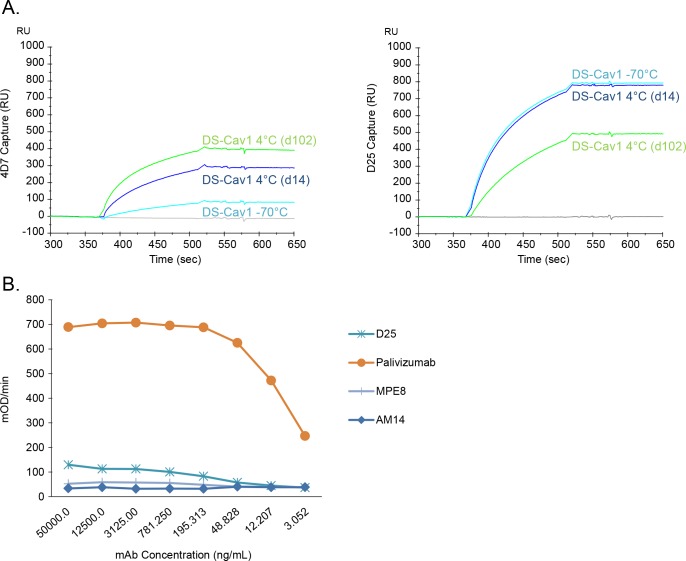
Monitoring DS-Cav1 stability with 4D7. (A) DS-Cav1 was stored at -70°C or at 4°C for either 14 or 102 days, and surface plasmon resonance was used to assess protein binding to 4D7 (left panel) and D25 (right panel). DS-Cav1 stored at -70°C and thawed immediately before use (cyan line) or stored at 4°C for 14 (blue line) or 102 (green line) days was flowed over the surface of 4D7- or D25-coated sensor chip channels, and response units over time, in seconds, were plotted. (B) Sandwich ELISA. DS-Cav1 stored for approximately 5 months at 4°C was captured on an ELISA plate coated with 4D7. The captured 4D7-reactive protein was bound by palivizumab (orange line), which recognizes both prefusion and postfusion forms of RSV F. In contrast, the captured 4D7-reactive protein was not bound by any of the prefusion-specific monoclonal antibodies tested, such as D25 (site Ø), MPE8 (site III), or AM14 (site V).

The data presented in Fig 3 reveal that a subset of the DS-Cav1 preparation was recognized by 4D7 and that this protein population was unable to bind D25. Reproducible results were obtained from multiple independently expressed and purified DS-Cav1 lots (data not shown). It was unclear whether a postfusion-like protein population, defined by the accessibility of the 4D7 epitope, was generated in cell culture and co-purified with the prefusion F protein or whether, despite the incorporation of prefusion-stabilizing mutations, a fraction of the molecules in the DS-Cav1 preparation had changed from a D25-binding prefusion conformation to an alternate form recognized by 4D7. To further explore the conformation-shift hypothesis, DS-Cav1 was stored frozen (-70°C) or at 4°C for 14 or 102 days, and binding to D25 and 4D7 was evaluated by SPR. After storage at 4°C for 14 days or 102 days, the 4D7 reactivity of DS-Cav1 increased significantly (Fig 4A). Concomitantly, prolonged storage of DS-Cav1 at 4°C resulted in a significant decrease in D25 binding (Fig 4A). Taken together, these results indicate that the conformation of DS-Cav1changes upon storage for prolonged periods of time at 4°C, and this change can be monitored by assessing the reactivity of the protein preparation to 4D7 and D25 antibodies.

To further understand whether the D25 and 4D7 antibody epitopes are present in a single molecule of RSV F stored at 4°C, we performed a sandwich ELISA assay as shown in Fig 4B. For this assay, 4D7 was used as the capture antibody, and 4°C-stored DS-Cav1 protein was applied to the 4D7-coated wells. Palivizumab was able to bind the 4D7-captured protein, indicating that at least a subset of the 4°C-stored DS-Cav1 bound 4D7, and the palivizumab epitope was accessible (Fig 4B). However, this protein did not react, or showed only a very low level of binding, to any of the prefusion-specific antibodies evaluated, including D25, MPE8 and AM14 (Fig 4B). These data suggest that the 4D7 and D25 epitopes are not both accessible on the same molecule and that the DS-Cav1 preparation may be a mixture of at least two antigen conformations, one that binds D25 but not 4D7 and one that binds 4D7 but not D25. Upon storage of DS-Cav1 for extended time at 4°C, the amount of D25-binding species decreases, while the amount of 4D7-binding species increases.

### Transmission electron microscopy (TEM) analysis

**Fig 5:**
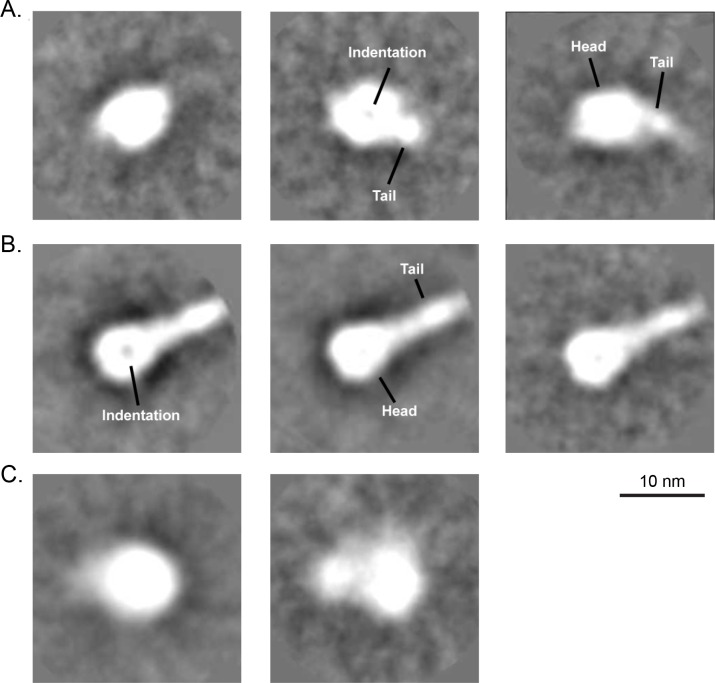
Analysis of RSV F proteins by negative stain transmission electron microscopy and 2D class averaging. (A) Representative averages for DS-Cav1 stored at -70°C and thawed immediately prior to analysis, indicating that mostly globular particles (left) or particles that contained a slightly tapered, short tail portion (middle and right) were observed. A characteristic indentation was visible in some of the head domains (middle). (B) Representative averages for postfusion RSV F protein stored at -70°C and thawed immediately prior to analysis, indicating that particles with distinct head and tail portions were primarily observed. A characteristic indentation was also visible in some of the head domains (left). (C) Representative averages for DS-Cav1 protein after long-term storage at 4°C. The lack of detail of the averages suggests that the conformation of 4°C-stored DS-Cav1 is more heterogeneous and does not resemble the postfusion form.

To directly visualize the potential DS-Cav1 conformational change, we used transmission electron microscopy (TEM) negative stain imaging with 2D class averaging analysis to evaluate DS-Cav1 stored at -70°C and thawed immediately prior to the analysis, DS-Cav1 stored at 4°C for approximately 3 months, and postfusion F protein. For DS-Cav1 stored frozen at -70°C, negative stain TEM with 2D averaging analysis showed that the protein preparation was primarily composed of particles that looked globular in shape and measured about ~8 nm (Fig 5A, left panel). Some of these particles appeared to have an indentation in the center (Fig 5A, middle panel), and some appeared to have a slightly longer tapered tail such that the particles measured 9–10 nm in length (Fig 5A, middle and right panel). It is possible that these are all different views of the same protein conformation. On the other hand, analysis of postfusion F revealed 14–20 nm particles with a distinct head and thinner tail portion (Fig 5B). The head of these particles was ~8 nm across and round in shape, and the tail was ~3 nm in width. The entire particle resembled a “lollipop” shape. In some particles, the head portion appeared to have an indentation in the center (Fig 5B, left panel), possibly a different view of the same protein conformation. These findings are consistent with the previously described X-ray crystallography studies of prefusion and postfusion RSV F proteins [13, 21].

We have shown that DS-Cav1, upon storage at 4°C, loses the ability to bind D25 and gains reactivity to the postfusion F-binding antibody, 4D7. Thus, we hypothesized that the 4°C- stored DS-Cav1 undergoes a conformational change from the prefusion structure. TEM with 2D class averaging showed that the 4°C-stored DS-Cav1 protein preparation contained mostly globular particles that appeared either as a single round ~ 8 nm shape or a structure containing two lobes, a larger ~8 nm lobe and a smaller ~5 nm lobe (Fig 5C). Both averages appeared to have slightly undefined borders, suggesting a high degree of heterogeneity in these preparations, as compared to those observed with analysis of freshly thawed DS-Cav1 or postfusion F protein. No species with the distinct “lollipop” shape prominently observed in the postfusion RSV F protein sample were detected, indicating that the 4°C-stored DS-Cav1 does not rearrange into the postfusion structure.

### Analysis of RSV F proteins by differential scanning fluorimetry

While the images of 4°C-stored DS-Cav1 were not as homogeneous as those of freshly thawed DS-Cav1, the TEM analysis showed that the 4°C-stored DS-Cav1 sample appears, grossly, like prefusion F. Although multiple conformations were not directly visualized, SPR and ELISA data indicate that the 4°C-stored DS-Cav1 preparation contains a mix of conformationally-distinct species that can be distinguished by their reactivity to 4D7 and D25. To further characterize these conformational changes, we performed thermal shift assays using label free differential scanning fluorimetry (DSF) [34]. The primary sequence of DS-Cav1 contains four tryptophan residues that allow monitoring of conformational transitions, subunit association, or denaturation events by measurement of intrinsic fluorescence [35]. DS-Cav1 tryptophan residues W27, W262, W290 and W481 are located in distinct regions of the protein (S3A Fig). W27 is part of the F2 domain, W262 and W290 are within the F1 domain, and W481 is located within the heterologous trimerization motif.

**Fig 6:**
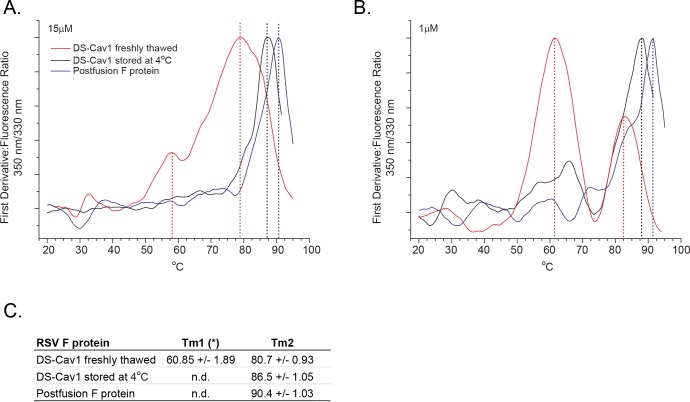
Analysis of RSV F proteins by differential scanning fluorimetry. First derivative of F350/F330 DSF unfolding curves for freshly thawed DS-Cav1 stored at -70°C (red line), DS-Cav1 stored for 90 days at 4°C (black line) and postfusion F protein (blue line). Transition midpoints are shown as vertical lines. (A) Protein concentration analyzed was 15 μM. (B) Protein concentration analyzed was 1 μM. (C) DSF transition midpoints of freshly thawed DS-Cav1, DS-Cav1 stored for 90 days at 4°C, and postfusion F. Mean values and standard deviations are calculated from measurements taken at protein concentrations between 35 μM and 0.27 μM. (*) Tm1 is observed only in preparations of freshly thawed DS-Cav1. The intensity of this transition increases with lower concentration.

Thermal unfolding curves between 20°C and 95°C were generated by calculating the emission ratio of fluorescence traces recorded at 350 nm and 330 nm. The fluorescence maximum of tryptophan molecules in an apolar environment is located at 330 nm, and with increasing exposure to an aqueous environment, the intensity component at 350 nm increases. The intensity ratio, F350/F330, best monitors conformational changes and/or unfolding of the protein structure as the protein preparation is heated [36]. DSF analysis was first performed at a protein concentration of 15 μM (Fig 6A), and the measurement revealed distinct melting temperatures (T_m_) for freshly thawed DS-Cav1, DS-Cav1 stored at 4°C for 90 days and postfusion F. At 90.4°C, the Tm of postfusion F was the highest of the three samples tested. The Tm of freshly thawed DS-Cav1 was the lowest at 80.7°C, and the Tm of the DS-Cav1 sample stored at 4°C was 86.5°C (Fig 6C).

The melting curve of freshly thawed -70°C-stored DS-Cav1 presented in Fig 6A showed an additional, less intense, transition at a lower temperature (58.7°C) that was not observed with postfusion F protein or DS-Cav1 stored at 4°C. To gain further insight into the nature of the structural differences between DS-Cav1stored at -70°C and DS-Cav1 stored at 4°C, DSF analyses were performed at protein concentrations between 0.3 μM and 35 μM (S3B Fig). At concentrations below 35 μM, the second, lower temperature transition (mean value of 60.85°C ± 1.89°C) was observed for freshly thawed DS-Cav1 but not for DS-Cav1 stored at 4°C or postfusion F protein (Fig 6B). The amplitude of this transition increased as the protein concentration decreased (S3B Fig), saturating below 1 μM with a transition midpoint at 2.8 μM (S3C Fig). Together, these data suggest that, with long-term storage at 4°C, the DS-Cav1 preparation adopts a conformation with increased thermal stability that is distinct from that of freshly thawed DS-Cav1 and postfusion F.

## Discussion

Developing a safe and effective vaccine to prevent RSV infection or disease is a high priority, and a number of vaccine candidates are currently under preclinical and clinical evaluation [37]. The majority of the RSV neutralizing antibody response in convalescent human serum is directed toward epitopes specific for the prefusion form of the RSV F surface glycoprotein, suggesting that a prefusion F-stabilized subunit vaccine, such as DS-Cav1, would elicit protective immunity against the disease [16, 20]. Rationally designed mutations stabilize DS-Cav1 in the prefusion conformation, and DS-Cav1 reactivity to the prefusion-specific, site Ø-binding, D25 antibody is largely maintained after incubation at 4°C for one week [20]. Recently, however, it was reported that prefusion-specific antibody binding to DS-Cav1 stored at 4°C for up to 50 days was reduced, indicating that its structural integrity was compromised [38]. Using a newly identified anti-RSV F antibody, 4D7, along with transmission electron microscopy and differential scanning fluorimetry, we sought to further characterize the long-term stability of DS-Cav1 at 4°C.

The 4D7 epitope was mapped to the previously defined antigenic site I using shotgun mutagenesis, and three residues on the F protein critical for 4D7 binding (V384, D385 and F387) were identified. Although the 4D7 binding site adopts a similar conformation in both the prefusion and postfusion RSV F structures, SPR experiments revealed that 4D7 binds to postfusion F and does not react to the site Ø-containing prefusion form of the protein. The 4D7 binding site appears to be fully exposed at the tip of postfusion F, while epitope availability may be restricted in the prefusion conformation where the 4D7 binding site is localized to the base of the head domain (Fig 1). Steric hindrance, rather than a difference in the epitope conformation between prefusion and postfusion F forms, may be the reason why many site I-reactive antibodies, like 4D7, bind specifically to postfusion F [18, 32, 33].

In addition to the primary critical residues already described, epitope mapping experiments also identified two secondary critical residues, R235 and P246 (data not shown). These residues are buried within the core of the postfusion F structure and, therefore, are unlikely to contact 4D7 directly [13]. In the postfusion, but not prefusion, F trimer structure, R235 residues form salt bridges with E232 residues on adjacent monomers [13, 21], likely contributing to the structural stability of postfusion F and enabling the binding of postfusion-reactive antibodies such as 4D7. Consistent with this interpretation, the R235A mutation reduced reactivity of another site I-binding postfusion-specific antibody, 3B1, to F protein but had no significant effect on binding of prefusion- specific monoclonal antibodies such as D25 [39].

In addition to binding the postfusion conformation of RSV F, we showed that 4D7 reacts to a subset of the protein found in preparations of purified DS-Cav1. This protein subset was unable to bind D25, indicating that the mix of protein conformations found in DS-Cav1 preparations could be distinguished by their ability to bind either 4D7 or D25. Furthermore, long-term storage of DS-Cav1 at 4°C led to an increase in 4D7-reactive forms with a parallel decrease in D25-reactivity. These data suggested that, over time at 4°C, the conformation of at least a subset of the DS-Cav1 protein shifts from a site Ø-containing prefusion conformation to a form in which the 4D7 epitope is exposed. Our experiments demonstrated that D25 and 4D7 epitopes are not both present on the same protein molecule; therefore, the 4°C-stored DS-Cav1 preparation must be a mixture of at least two different protein isoforms.

Transmission electron microscopy was unable to distinguish multiple protein conformations in the DS-Cav1 sample stored long-term at 4°C, although the 4°C-stored protein appeared more heterogeneous than that thawed immediately prior to analysis. However, it was clear from the TEM imaging that the 4°C-stored DS-Cav1 protein did not adopt a postfusion-like structure, likely because the prefusion-stabilizing mutations introduced into DS-Cav1 lock the head domain and prevent the large conformational rearrangement required to adopt the postfusion-like “lollipop” structure [20].

In contrast, analysis by label free differential scanning fluorimetry was able to biophysically distinguish the 4°C-stored DS-Cav1 from both freshly thawed DS-Cav1 and postfusion F. The intermediate melting temperature measured for DS-Cav1 stored at 4°C suggested that, upon storage at 4°C, DS-Cav1 shifts to a more thermostable conformation. SPR and ELISA analysis suggested that at least two protein isoforms were present in the preparation of DS-Cav1 stored long-term at 4°C. However, only a single Tm was observed when these samples were analyzed by DSF. Given the breadth of the transition peaks measured for the RSV F preparations, and the overlap observed, there may not be sufficient resolution to distinguish distinct isoforms within the 4°C-stored DS-Cav1 sample. Alternatively, it’s possible that the majority of the DS-Cav1 preparation, after storage at 4°C for 3 months, had adopted some number of alternate conformations, all of which showed a similar increase in thermostability.

Relative to that of postfusion F or DS-Cav1 stored at 4°C, the unfolding transition of freshly thawed DS-Cav1 was broad, spanning over 30°C (Fig 6A). While narrow unfolding transitions, such as those observed for postfusion F or 4°C-stored DS-Cav1, are indicative of more rigid structures [40], the broad transition observed for the freshly thawed DS-Cav1 sample suggests that there is conformational flexibility in the prefusion-like structure. This is consistent with the atomic mobility described previously for DS-Cav1 [20].

The thermal melting curve of freshly thawed 15 μM DS-Cav1, but not 4°C-stored DS-Cav1 or postfusion F, revealed a second, less pronounced, transition temperature with at Tm of approximately 60°C (Fig 6A and 6C). The contribution of this early transition increased as the concentration of protein decreased (S3A Fig). Since monomer in the monomer:trimer equilibrium becomes more abundant at low protein concentration [41], one possible explanation for this early, low temperature transition is that it represents unfolding of monomeric, rather than trimeric, DS-Cav1. The thermal melting curve of 1 μM 4°C-stored DS-Cav1 may also show a similar early transition (Fig 6B), again suggesting that, while the DS-Cav1 stored at 4°C is more stable than freshly thawed DS-Cav1, it’s likely not as stable as postfusion F, for which no second transition temperature was detected at any of the protein concentrations tested. This may not be surprising since the prefusion-stabilizing mutations that define DS-Cav1 prevent the protein from converting into the low energy conformation adopted by postfusion F.

In conclusion, these data highlight the utility of the newly identified 4D7 mouse monoclonal antibody as a reagent for monitoring conformational changes of DS-Cav1 and potentially other stabilized prefusion F antigens that occur with prolonged storage at 4°C. The data also describe the existence of at least, but not limited to, two distinct DS-Cav1 isoforms, one that binds the prefusion-specific D25 antibody but not 4D7 and another that binds 4D7 but not D25. Upon storage at 4°C, the amount of D25-binding isoforms decreases, consistent with previously published results showing that DS-Cav1 stored at 4°C is less reactive over time to another prefusion-specific antibody, CR9501 [38]. In parallel to the observed decrease in prefusion-like conformation, the amount of 4D7-reactive forms increase. This increase, however, is not caused by a shift to a postfusion-like conformation, but to an alternate structure that is more thermostable than the prefusion form.

It’s not clear if the biophysical changes described herein would diminish the functional immune response provided by the vaccine antigen. However, the superior potency of site Ø-directed monoclonal antibodies suggests that a reduction in the amount of site Ø-containing F protein might have a negative effect on vaccine efficacy [20]. Thus, exploring ways to further stabilize DS-Cav1 is of interest, either through formulation, with the addition of protein-stabilizing excipients, or by rational design, with the addition of other prefusion-stabilizing mutations, particularly those that would further stabilize regions near antigenic site Ø and site I, shown herein to be detectably altered by long-term storage at 4°C. Moreover, 4D7 could be used to isolate and further characterize particular subsets of DS-Cav1 protein, providing an understanding that would aid in the rational design of prefusion F molecules with improved stability.

## Supporting Information

S1 Fig Amino acid sequence of mAb 4D7 variable regions. (PDF)

S2 Fig Assessing 4D7:antigen complex binding to D25. DS-Cav1 was pre-incubated with 4D7 (blue line, left panel), D25 (blue line, right panel) or buffer (red line) before the antibody:antigen complex was flowed over the surface of a D25-coated sensor chip. Response units were plotted over time, in seconds.
(PDF)

S3 Fig Differential scanning fluorimetry analysis. (A) Ribbon representation of the DS-Cav1 monomer backbone. The F2 fragment is colored in blue, the F1 fragment in red and the foldon trimerization motif in green. Tryptophan residues W27, W262, W290 and W481 are shown in space filling models. (B) F350/F330 DSF unfolding curves for freshly thawed DS-Cav1recorded at 35 μM, 17.5 μM, 4.4 μM (duplicates), 2.2 μM, 1.1 μM, 0.5 μM and 0.3 μM protein concentrations. Transition midpoints are located at 60.85°C and 80.7°C. The intensity of the transition centered at 60.85°C increases with lower protein concentration. (C) The integral between 50°C and 75°C (area of Tm1) of F350/F330 DSF unfolding curves for freshly thawed DS-Cav1 at 35 μM, 17.5 μM, 4.4 μM, 2.2 μM, 1.1 μM, 0.5 μM and 0.3 μM was plotted against the protein concentration. The data points are fitted with a sigmoidal curve. The midpoint of the sigmoidal curve is at 2.8 μM.
(PDF)
