# Supplementary material for: Stability Characterization of a Vaccine Antigen Based on the Respiratory Syncytial Virus Fusion Glycoprotein
Source: PLoS One. 2016 Oct 20;11(10):e0164789. doi: 10.1371/journal.pone.0164789 (PMC5072732; doi:10.1371/journal.pone.0164789)
Supplement: S1 Fig — (PDF) [file pone.0164789.s001.pdf]

Heavy Chain Variable Region:
QVHLQQSGAELVKPGASVKLSCKASGYTFTSYYMYWVKQRPGQGLEWIGEIIPSMGSTNLNEKFKRKATL
TADKSSSTAYMQLSSLTSEDSAVYYCIRGGYYGYYWYFDVWGAGTTVTVSS

Light Chain Variable Region:
DIVLTQSPASLAVSLGQRATISCRASESVDNYGISFMNWFQQKPGQPPKLLIYAASNQGSGVPARFSGSGSG
TDFSLNIHPMEEDDTAMYFCQQSKEVPRTFGGGTKLEIK
