# Supplementary figures and images for: Stability Characterization of a Vaccine Antigen Based on the Respiratory Syncytial Virus Fusion Glycoprotein
Source: PLoS One. 2016 Oct 20;11(10):e0164789. doi: 10.1371/journal.pone.0164789 (PMC5072732; doi:10.1371/journal.pone.0164789)

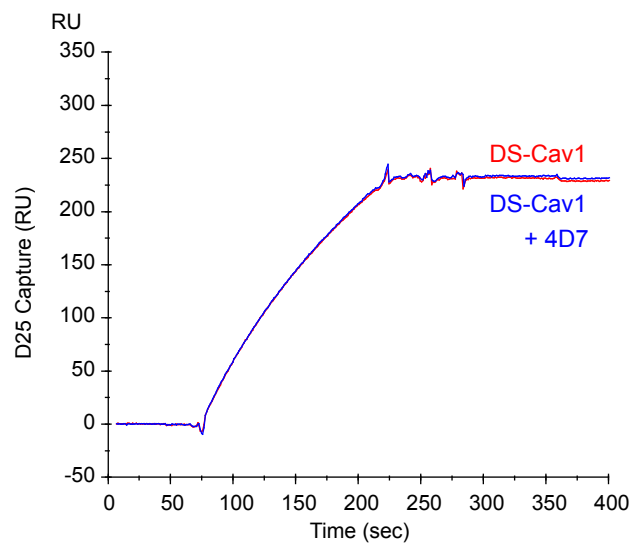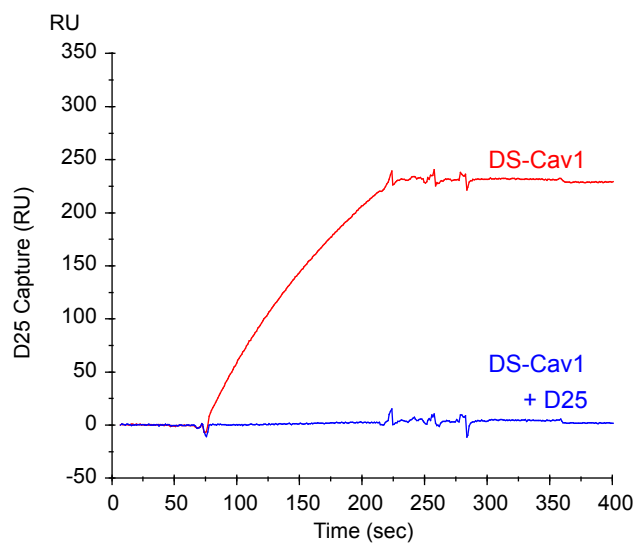

Supplement: S2 Fig — DS-Cav1 was pre-incubated with 4D7 (blue line, left panel), D25 (blue line, right panel) or buffer (red line) before the antibody:antigen complex was flowed over the surface of a D25-coated sensor chip. Response units were plotted over time, in seconds. (PDF) [file pone.0164789.s002.pdf]

A.

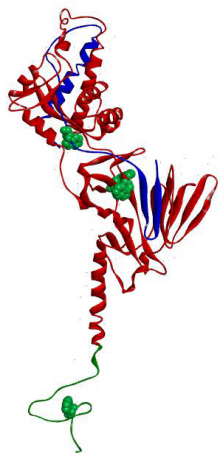

B.

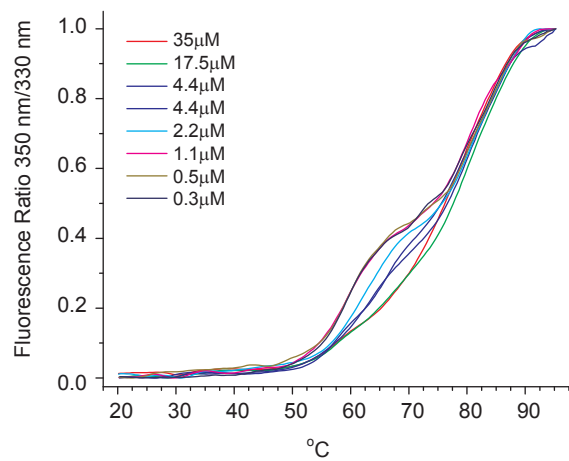

C.

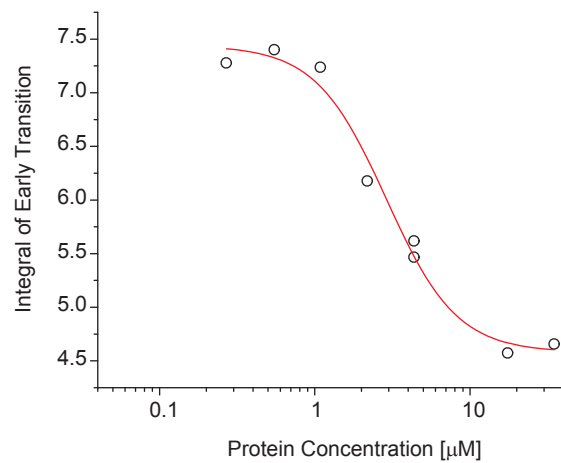

Supplement: S3 Fig — (A) Ribbon representation of the DS-Cav1 monomer backbone. The F2 fragment is colored in blue, the F1 fragment in red and the foldon trimerization motif in green. Tryptophan residues W27, W262, W290 and W481 are shown in space filling models. (B) F350/F330 DSF unfolding curves for freshly thawed DS-Cav1recorded at 35 μM, 17.5 μM, 4.4 μM (duplicates), 2.2 μM, 1.1 μM, 0.5 μM and 0.3 μM protein concentrations. Transition midpoints are located at 60.85°C and 80.7°C. The intensity of the transition centered at 60.85°C increases with lower protein concentration. (C) The integral between 50°C and 75°C (area of Tm1) of F350/F330 DSF unfolding curves for freshly thawed DS-Cav1 at 35 μM, 17.5 μM, 4.4 μM, 2.2 μM, 1.1 μM, 0.5 μM and 0.3 μM was plotted against the protein concentration. The data points are fitted with a sigmoidal curve. The midpoint of the sigmoidal curve is at 2.8 μM. (PDF) [file pone.0164789.s003.pdf]
